# Prognostic value of microRNAs in osteosarcoma: a meta-analysis

**DOI:** 10.18632/oncotarget.14429

**Published:** 2017-01-02

**Authors:** Yun Hak Kim, Tae Sik Goh, Chi-Seung Lee, Sae Ock Oh, Jeung Il Kim, Seung Hyeon Jeung, Kyoungjune Pak

**Affiliations:** ^1^ BEER, Busan Society of Evidence-Based Medicine and Research, Busan, Republic of Korea; ^2^ Department of Anatomy, School of Medicine, Pusan National University, Yangsan, Gyeongnam, Republic of Korea; ^3^ Department of Orthopaedic Surgery and Biomedical Research Institute, Pusan National University Hospital, Busan, Republic of Korea; ^4^ Biomedical Research Institute, Pusan National University Hospital and School of Medicine, Pusan National University, Busan, Republic of Korea; ^5^ Department of Nuclear Medicine and Biomedical Research Institute, Pusan National University Hospital, Busan, Republic of Korea

**Keywords:** microRNA, osteosarcoma, prognosis, meta-analysis

## Abstract

**BACKGROUND:**

Osteosarcoma is the most common primary bone malignancy. We meta-analyzed the prognostic value of altered miRNAs in patients with osteosarcoma.

**METHODS:**

Sources from MEDLINE (from inception to August 2016) and EMBASE (from inception to August 2016) were searched. Studies of osteosarcoma with results of miRNA and studies that reported survival data were included and two authors performed the data extraction independently. Any discrepancies were resolved by a consensus. The outcome was overall survival and event-free survival assessed using hazard ratios (HRs).

**RESULTS:**

After reviewing the full text of 65 articles, 25 studies including 2,278 patients were eligible in this study. The pooled HR for deaths was 1.40 (95% confidence interval [CI] 1.01-1.94, *p*=0.04) with random-effects model (χ^2^=113.08, *p*<0.00001, I^2^=79%) for patients of osteosarcoma with lower expression of miRNA. However, the pooled HR for events was not significant (HR 0.97, 0.63-1.48, *p*=0.87, χ^2^=72.65, *p*<0.00001, I^2^=79%). In pathway analysis of miRNAs, miRNA449a, 199-5p, 542-5p have common target genes.

**CONCLUSIONS:**

Expression level of miRNA in patients of osteosarcoma is important as a prognostic factor.

## INTRODUCTION

Osteosarcoma is the most common primary bony malignancies and first leading cause of sarcoma-related deaths in children and young adults. It mainly occurs in metaphyseal area of distal femur and proximal tibia. Osteosarcoma is highly aggressive tumor and most likely metastasizes to the lung [1]. Even though the survival rate has modestly increased over the last 2 decades by implicating radiotherapy and neo-adjuvant chemotherapy, five-year survival rate of metastatic osteosarcoma still remains in the range of 15% to 30%. For localized osteosarcoma which can be totally resectable, the five-year survival rate increases up to 70% [2]. Hence, excavating novel prognostic biomarkers of osteosarcoma is strongly needed to detect tumor at early stage. They will contribute to select better treatment options in earlier stages and predict the outcome more accurately.

MicroRNAs (miRNAs) are a class of small noncoding RNAs, which could interfere translation of many proteins after gene transcription [3]. Previous studies revealed that they are implicated on a variety of physiological processes including cellular differentiation, apoptosis, angiogenesis and cell proliferation [4]. Additionally, miRNAs play a pivotal role in tumorigenesis that can act either as tumor suppressor genes or oncogenes [3, 5]. Level of miRNAs expressed in several malignancies, including lung, kidney, liver, and cervical cancer were significantly different from that of normal tissue [6, 7]. Recent researches have demonstrated that various miRNAs are also altered in osteosarcoma tissue or blood sample [8]. These discoveries may indicate that miRNA could be potential prognostic biomarkers of osteosarcoma. Therefore, we meta-analyzed the prognostic value of altered miRNAs in patients with osteosarcoma.

## RESULTS

### Study characteristics

**Figure 1:**
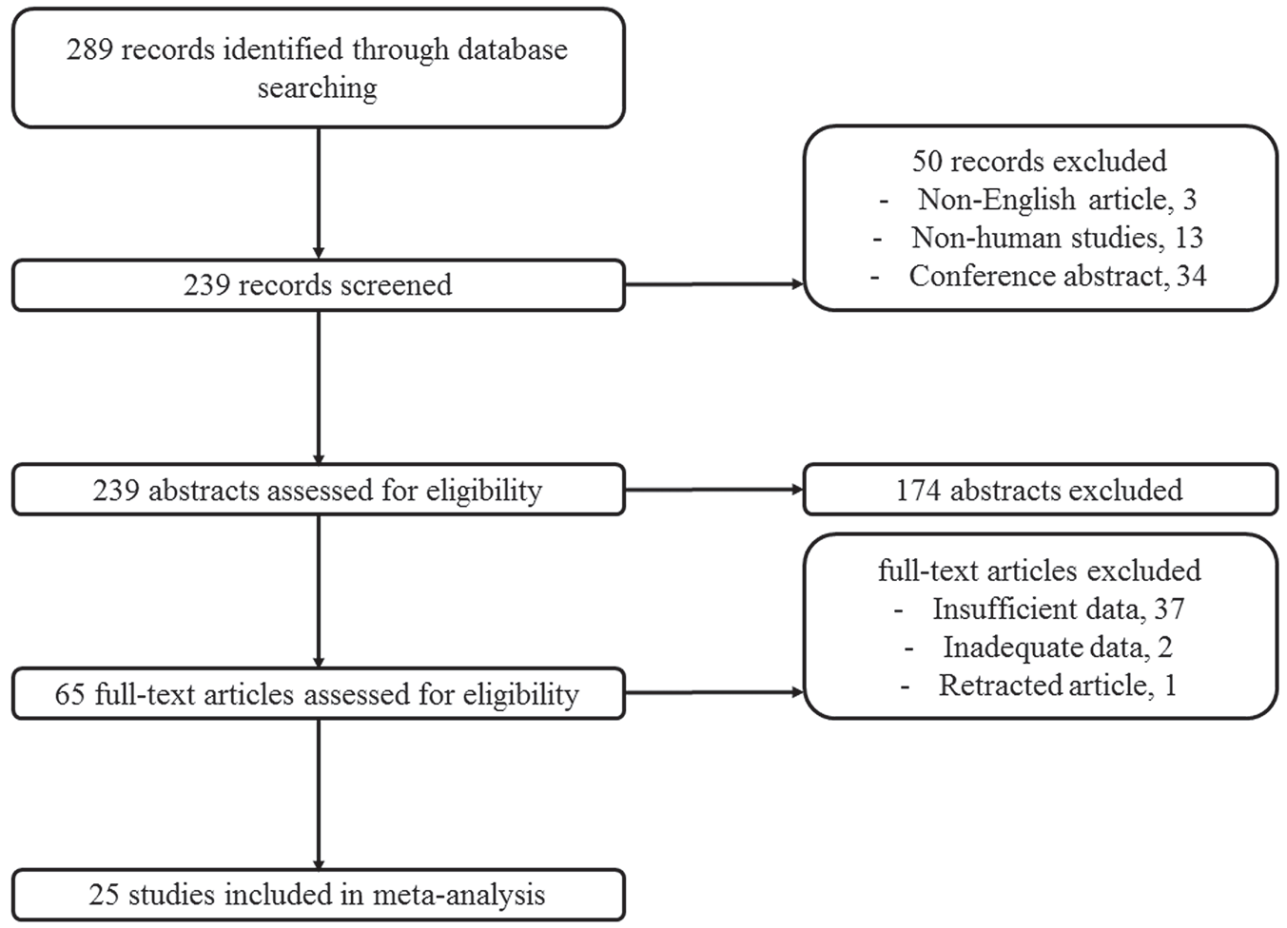
Flow chart

**Figure 2:**
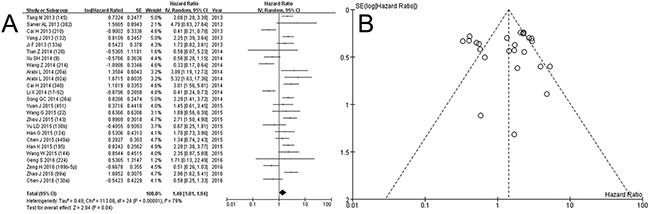
Forest plot A. and funnel plot B. for deaths of low expression of miRNA in osteosarcoma

**Figure 3:**
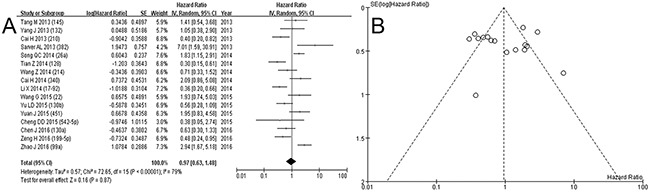
Forest plot A. and funnel plot B. for events of low expression of miRNA in osteosarcoma

The electronic search identified 289 articles. Non-English language articles (n=3), non-human studies (n=13), conference abstracts (n=34), and 174 studies that did not meet the inclusion criteria based on their title and abstract were excluded. After reviewing the full text of 65 articles, 25 studies including 2,278 patients were eligible for inclusion in the study. The detailed procedure is shown in Figure 1. The studies included in this meta-analysis reported the prognostic value of 26 miRNAs in patients with osteosarcoma. Twenty-five studies assessing 26 miRNA expressions were included in the meta-analysis [9–33]. The prognostic value of miRNA expressions was assessed by analyzing overall survival (OS) in 2,278 patients, event free survival (EFS) in 1,569 patients. Visual inspection of the funnel plot suggested no evidence of publication bias (Figure 2B and 3B). The study characteristics are summarized in Table 1.

**Table 1:** Studies included in this meta-analysis

Author	Year of publication	Country	miRNA	No. of patients	Stage	Follow-up (months)	Endpoints	Expression associates with poor prognosis	Assay method	Sample
Cai H [10]	2013	China	210	92	-	105^a^	OS, EFS	High	RT-PCR	Tissue
Li X [19]	2014	China	17-92 cluster	117	I-III	55^a^	OS, EFS	High	RT-PCR	Tissue
Tian Z [23]	2014	China	128	100	-	38.89^a^	OS, EFS	High	RT-PCR	Tissue
Xu SH [27]	2014	China	9	79	I-III	60^a^	OS	High	RT-PCR	Tissue
Wang Z [26]	2014	China	214	92	-	107^a^	OS, EFS	High	RT-PCR	Tissue
Cheng DD [14]	2015	China	542-5p	40	-	40	EFS	High	RT-PCR	Tissue
Yu LD [29]	2015	China	130b	68	I-III	36	OS, EFS	High	RT-PCR	Tissue
Chen J [12]	2016	China	130a	86	I-IV	60^a^	OS, EFS	High	RT-PCR	Tissue
Zeng H [31]	2016	China	199b-5p	98	I-IV	60^b^	OS, EFS	High	RT-PCR	Tissue
Yang J [28]	2013	China	132	166	II-III	105^a^	OS, EFS	Low	RT-PCR	Tissue
Tang M [22]	2013	China	145	166	II-III	100^a^	OS, EFS	Low	RT-PCR	Tissue
Sarver AL [20]	2013	USA	382	8	-	194^a^	OS, EFS	Low	RT-PCR	Tissue
Ji F [18]	2013	China	133a	92	I-III	60^a^	OS	Low	RT-PCR	Tissue
Song QC [21]	2014	China	26a	144	II-III	140^a^	OS, EFS	Low	RT-PCR	Tissue
Cai H [11]	2014	China	340	92	-	100^a^	OS, EFS	Low	RT-PCR	Tissue
Arabi L [9]	2014	Switzerland	20a	57	-	112	OS	Low	RT-PCR	Tissue
Arabi L [9]	2014	Switzerland	92a	57	-	112	OS	Low	RT-PCR	Tissue
Zhou J [33]	2015	China	143	45	-	36^c^	OS	Low	RT-PCR	Tissue
Yuan J [30]	2015	China	451	118	II-III	80^a^	OS, EFS	Low	RT-PCR	Tissue
Wang W [25]	2015	China	144	67	I-IV	80^a^	OS	Low	RT-PCR	Tissue
Wang G [24]	2015	China	22	52	I-IV	60c	OS, EFS	Low	RT-PCR	Tissue
Han K [17]	2015	China	195	107	II-III	90^a^	OS	Low	RT-PCR	Tissue
Han G [16]	2015	China	124	105	II-III	80^a^	OS	Low	RT-PCR	Tissue
Chen J [13]	2015	China	449a	60	I-III	60^b^	OS	Low	RT-PCR	Tissue
Zhao J [32]	2016	China	99a	130	II-III	60^a^	OS, EFS	Low	RT-PCR	Tissue
Geng S [15]	2016	China	224	40	-	60	OS	Low	RT-PCR	Tissue

- Follow-up; ^a^median, ^b^heterogeneity of logrank test, ^c^mean

- OS, overall survival; EFS, event free survival; RT-PCR, reverse transcription polymerase chain reaction

### miRNA expression and prognosis of osteosarcoma

To analyze the prognostic value of low expression of miRNA in osteosarcoma, forest plots with OS and EFS are depicted in Figure 2 and 3. The pooled hazard ratio (HR) for deaths was 1.40 (95% confidence interval [CI] 1.01-1.94, *p*=0.04) with random-effects model (χ^2^=113.08, *p*<0.00001, I^2^=79%), which means more deaths with lower expression of miRNA in osteosarcoma. However, the pooled HR for events did not show the significant value (HR 0.97, 0.63-1.48, *p*=0.87, χ^2^=72.65, *p*<0.00001, I^2^=79%). Due to heterogeneity in pooled analysis of miRNA, we performed a subgroup analysis according to the expression level of miRNA. The HRs were calculated on the basis of low expression of miRNA, which means HR > 1 and < 1 implied poor and good prognosis for patients with low miRNA expression.

### A. Worse prognosis with high miRNA expression

**Figure 4:**
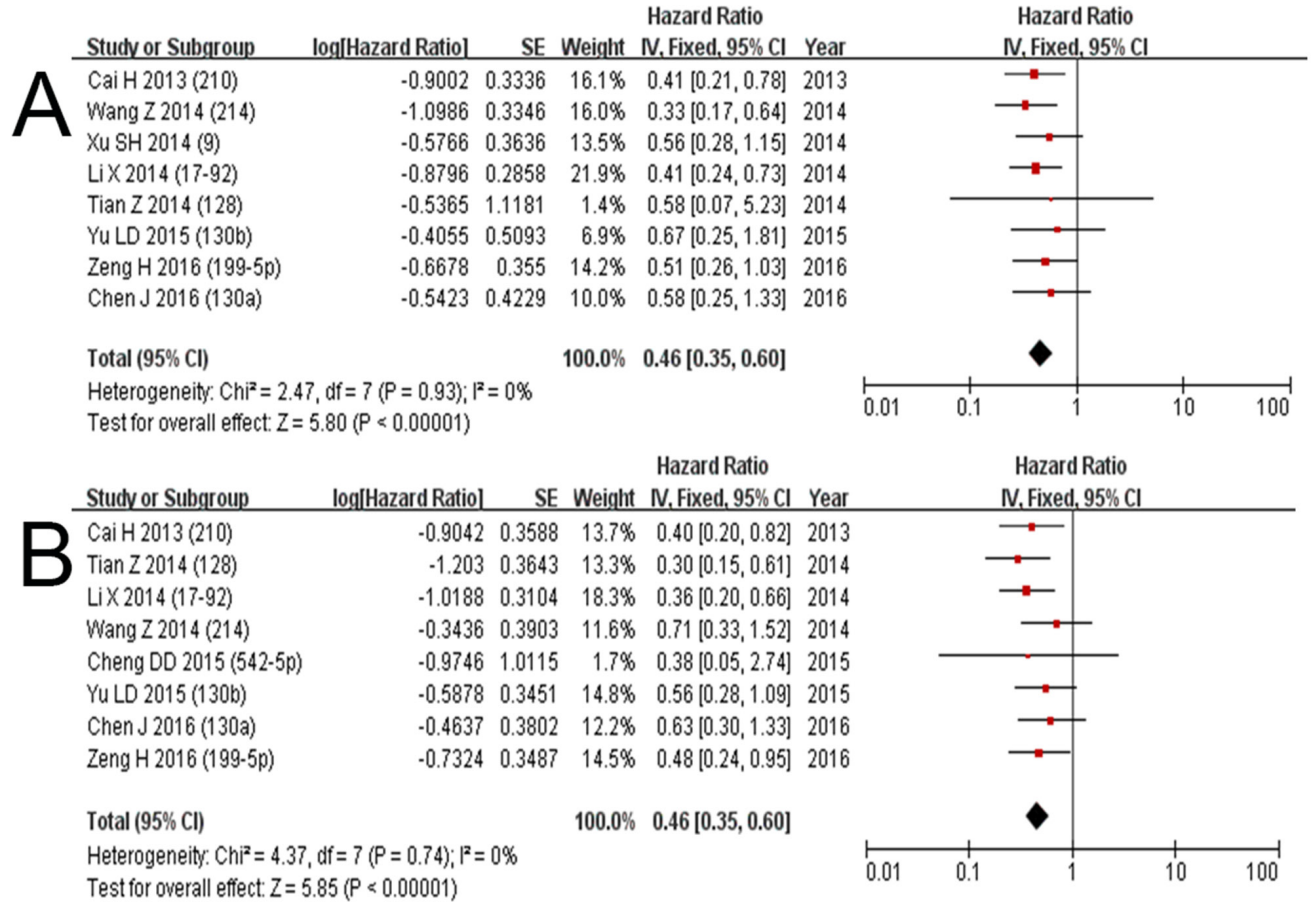
Subgroup analysis: worse prognosis with high expression of miRNA; forest plots for deaths A. and events B. of low expression of miRNA in osteosarcoma

High expressions of miRNA210, 17-92 cluster, 128, 9, 214, 542-5p, 130b, 130a, 199b-5p were associated with poor prognosis of osteosarcoma patients, [10, 12, 14, 19, 23, 26, 27, 29, 31]. Eight studies were included to analyze OS with miRNA210, 17-92 cluster, 128, 9, 214, 130b, 130a, 199-5p in 732 patients [10, 12, 19, 23, 26, 27, 29, 31]. The HR ranged between 0.33 and 0.67 with a pooled HR for deaths of 0.45 (0.35-0.60, *p*<0.00001) with low expression of miRNA, and the test for heterogeneity gave no significant results (χ^2^=2.47, *p*=0.93, I^2^=0%) (Table 2). The EFS was analyzed with miRNA210, 17-92 cluster, 128, 214, 542-5p, 130b, 130a, 199b-5p based on 8 studies including 693 patients [10, 12, 14, 19, 23, 26, 29, 31]. The pooled HR of high miRNA for adverse events was 0.46 with a range from 0.30 to 0.71 (0.35-0.60, *p*<0.00001), and the test for heterogeneity gave no significant results (χ^2^=4.37, *p*=0.74, I^2^=0%) (Table 2). The forest plots for OS and EFS of high miRNA with worse prognosis are shown in Figure 4.

**Table 2:** Subgroup analysis according to miRNA expression with prognosis

	No. of studies	miRNA	HR	95% CI of HR	Heterogeneity I^2^ (%)	Model used
Worse prognosis with high miRNA expression						
OS	8	210, 17-92, 128, 9, 214, 130b, 130a, 199b-5p	0.46	0.35-0.60	0	Fixed effect
EFS	8	210, 17-92, 128, 214, 542-5p, 130b, 130a, 199b-5p	0.46	0.35-0.60	0	Fixed effect
Worse prognosis with low miRNA expression						
OS	16	132, 145, 382, 133a, 26a, 340, 20a, 92a, 143, 451, 144, 22, 195, 124, 449a, 99a, 224	2.25	1.90-2.66	0	Fixed effect
EFS	8	132, 145, 382, 26a, 340, 451, 22, 99a	2.05	1.67-5.18	0	Fixed effect

- HR, hazard ratio; OS, overall survival; EFS, event free survival

### B. Worse prognosis with low miRNA expression

**Figure 5:**
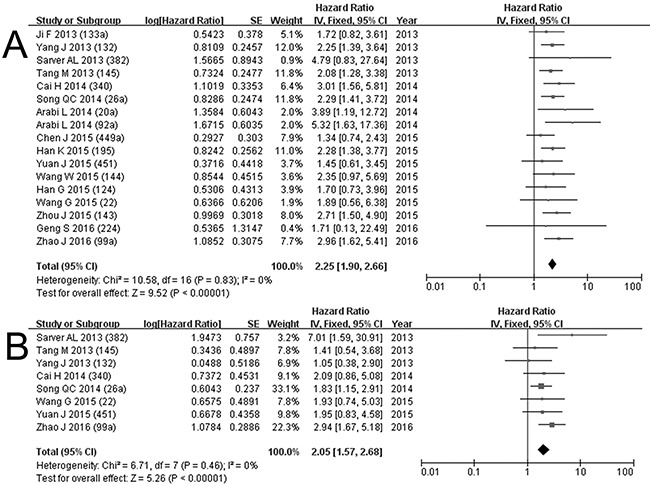
Subgroup analysis: worse prognosis with low expression of miRNA; forest plots for deaths A. and events B. of low expression of miRNA in osteosarcoma

Low expression of miRNA132, 145, 382, 133a, 26a, 340, 20a, 92a, 143, 451, 144, 22, 195, 124, 449a, 99a, 224 were found to be associated with a poor prognosis [9, 11, 13, 15–18, 20–22, 24, 25, 28, 30, 32, 33]. Sixteen studies analyzed OS with miRNA132, 145, 382, 133a, 26a, 340, 20a, 92a, 143, 451, 144, 22, 195, 124, 449a, 99a, 224 in 1,506 patients [9, 11, 13, 15–18, 20–22, 24, 25, 28, 30, 32, 33], and eight studies (miRNA132, 145, 382, 26a, 340, 451, 22, 99a) containing 876 patients that reported EFS were included to calculate the combined HRs [11, 20–22, 24, 28, 30, 32]. The HR for deaths ranged widely between 1.34 and 5.32 with a combined HR of 2.25 (1.90-2.66, *p*<0.00001), and the pooled HR for events was 2.05 with a range from 1.05 to 7.01 (1.57-2.68, *p*<0.00001). There was no significant heterogeneity (OS: χ^2^=10.58, p=0.83, I^2^=0%, EFS: χ^2^=6.71, *p*=0.46, I^2^=0%). The forest plots for OS and EFS of low miRNA with worse prognosis are shown in Figure 5.

### C. Pathway analysis of miRNAs

**Figure 6:**
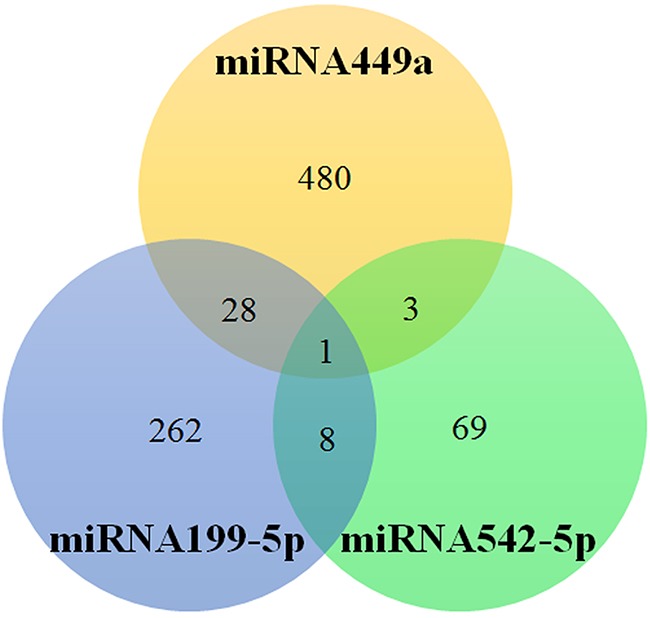
The number of target genes of miRNA449a, 199-5p and 542-5p

**Figure 7:**
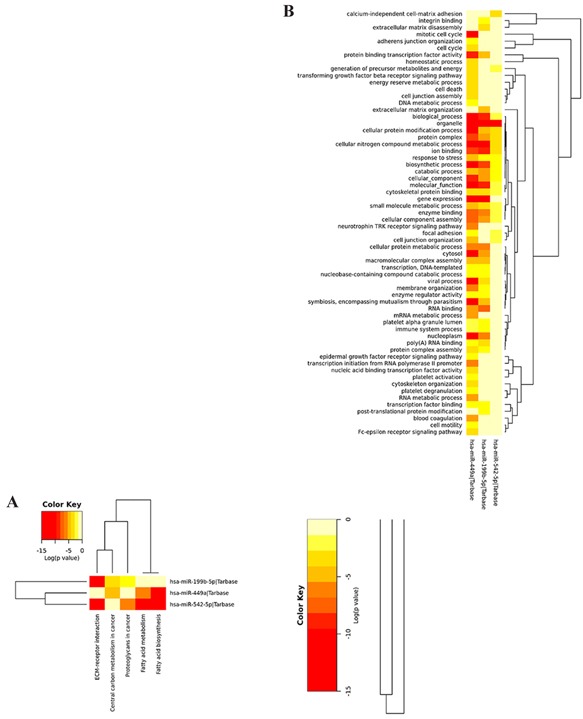
Heatmap of pathway analysis of miRNA449a, 199-5p and 542-5p by KEGG pathway A. and GO categories B

In a pathway analysis of miRNAs using mirPath v3.0 based on Tarbase, KEGG and GO, miRNA449a, 199-5p, 542-5p have target genes and signaling pathways, but others do not have known targets (Figure 6 and 7, Table 3) [34].

**Table 3:** pathway analysis of miRNA449a, 199b-5p, 542-5p

pathway analysis	*p*	no. of target genes	miRNA -449a	miRNA- 199b-5p	miRNA- 542-5p
**KEGG pathway**					
Fatty acid biosynthesis	0	3	Y	N	Y
Fatty acid metabolism	0	3	Y	N	Y
ECM-receptor interaction	0	10	N	Y	Y
Proteoglycans in cancer	2.47E-07	15	Y	Y	N
Central carbon metabolism in cancer	1.58E-06	14	Y	Y	N
**GO Category**					
molecular function	0	789	Y	Y	Y
cellular protein modification process	0	155	Y	Y	Y
biological process	0	780	Y	Y	Y
biosynthetic process	0	255	Y	Y	Y
cellular nitrogen compound metabolic process	0	299	Y	Y	Y
ion binding	0	317	Y	Y	Y
organelle	0	596	Y	Y	Y
protein complex	1.33E-15	232	Y	Y	Y
cellular component	5.00E-15	784	Y	Y	Y
enzyme binding	2.80E-13	103	Y	Y	Y
cellular component assembly	5.09E-12	102	Y	Y	Y
catabolic process	2.86E-10	126	Y	Y	Y
response to stress	4.90E-08	136	Y	Y	Y
cytoskeletal protein binding	1.68E-07	61	Y	Y	Y
small molecule metabolic process	2.90E-07	130	Y	Y	Y
gene expression	0	70	Y	Y	N
symbiosis, encompassing mutualism through parasitism	0	58	Y	Y	N
nucleoplasm	2.22E-16	97	Y	Y	N
cytosol	1.89E-15	174	Y	Y	N
viral process	1.43E-14	50	Y	Y	N
RNA binding	2.72E-12	122	Y	Y	N
cellular protein metabolic process	4.33E-12	43	Y	Y	N
protein binding transcription factor activity	6.72E-12	49	Y	Y	N
macromolecular complex assembly	1.36E-08	64	Y	Y	N
membrane organization	9.90E-08	45	Y	Y	N
cell junction organization	5.04E-06	18	Y	N	Y
poly(A) RNA binding	5.87E-06	103	Y	Y	N
protein complex assembly	2.17E-05	52	Y	Y	N
transcription, DNA-templated	9.55E-05	127	Y	Y	N
nucleobase-containing compound catabolic process	0.000168	53	Y	Y	N
enzyme regulator activity	0.000207	51	Y	Y	N
transcription factor binding	0.000255	43	Y	Y	N
generation of precursor metabolites and energy	0.0004	20	Y	N	Y
focal adhesion	0.000601	35	Y	N	Y
immune system process	0.00162	81	Y	Y	N
activation of signaling protein activity involved in unfolded protein response	0.002297	9	Y	Y	N
platelet alpha granule lumen	0.003495	7	Y	Y	N

## DISCUSSION

This study evaluated the prognostic value of miRNA in patients with osteosarcoma. As we described above, the combined HR of OS from the included studies was 1.40 (1.01-1.94, *p*=0.04), which provided that low expression of miRNAs was related with shorter OS in patients with osteosarcoma. However, the combined HR of EFS was not significant. Due to heterogeneity in pooled analysis of miRNA, we performed a subgroup analysis according to the expression level of miRNA. For a more detailed analysis, we performed a subgroup analysis according to miRNA expressions (high or low) related with poor prognosis. High expression of miRNA 210, 17-92 cluster, 128, 9, 214, 542-5p, 130b, 130a, and 199b-5p and low expression of miRNA 132, 145, 382, 133a, 26a, 340, 20a, 92a, 143, 451, 144, 22, 195, 124, 449a, 99a, 224 were associated with a poor prognosis of either deaths or events.

The possible role of miRNAs during osteosarcomagenesis is also investigated. Altered or aberrant expression of miRNA, its function during tumorigenesis, and potential target genes are summarized in Table 4. Nine miRNAs (miRNA 210, 17-92 cluster, 128, 9, 214, 542-5p, 130b, 130a, and 199b-5p) whose expression level correlated with poor prognosis function as an oncogene in osteosarcoma. Of that, miRNA 210 contributes its action in tumor initiation. miRNA 17-92 cluster, 214, 130b have been reported as they progress early stage osteosarcoma into advanced stage. And miRNA 9, 130a are associated with metastasis. On the contrary, 17 miRNAs (132, 145, 382, 133a, 26a, 340, 20a, 92a, 143, 451, 144, 22, 195, 124, 449a, 99a, 224) function as a tumor suppressor gene, which expressed low level when they were related with worse outcome. Among these miRNAs, miRNA 382, 133a, 26a, 340, 143, 144, 195 have shown an anti-metastatic activity. Especially, miRNA 133a, 143 could be utilized as a future therapeutic target for lung metastasis. Moreover, miRNA 132, 145, 92a might act as a therapeutic standard for chemotherapy.

**Table 4:** miRNAs with relative expression level associated with poor prognosis, their function and target or related gene

miRNA	Expression level associate with poor prognosis	Function	Target or related gene
miR-210[10]	high	Oncogene (Tumor Initiation)	N/D
miR-17–92[19]	high	Oncogene (Tumor Progression)	N/D
miR-128[23]	high	Oncogene	Inhibits PTEN
miR-9[27]	high	Oncogene Promote metastasis	N/D
miR-214[26]	high	Oncogene (Tumor Progression)	N/D
miR-542-5p [14]	high	Oncogene	Inhibits HUWE1
miR-130b [29]	high	Oncogene (Tumor Progression)	Inhibits PPARγ
miR-130a [12]	high	Oncogene Promote metastasis	Inhibits PTEN
miR-199b-5p [31]	high	Oncogene	HES1
miR-132 [28]	low	Discriminate good responders from poor responders	N/D
miR-145 [22]	low	Tumor suppressor Discriminate good responders from poor responders	N/D
miR-382 [20]	low	Oncogene Suppress metastasis	Stabilize MYC
miR-133a [18]	low	Tumor suppressor Suppress pulmonary metastasis	Inhibits Bcl-xL, Mcl-1
miR-26a [21]	low	Tumor suppressor Suppress metastasis and recurrence	Inhibits EZH2
miR-340 [11]	low	Tumor suppressor Suppress metastasis	N/D
miR-20a [9]	low	Tumor suppressor	Inhibits FAS
miR-92a [9]	low	Discriminate ifosfamide responder	Inhibits FAS
miR-143 [33]	low	Tumor suppressor Suppress pulmonary metastasis	MMP-13 Bcl-2
miR-451 [30]	low	Tumor suppressor	N/D
miR-144 [25]	low	Tumor suppressor Suppress metastasis	Inhibits ROCK1, ROCK2
miR-22 [24]	low	Tumor suppressor	Inhibits HMGB1
miR-195 [17]	low	Suppress metastasis	Inhibits CCND1
miR-124 [16]	low	Tumor suppressor	N/D
miR-449a [13]	low	Tumor suppressor	Inhibits Bcl-2
miR-99a [32]	low	Tumor suppressor	mTOR
miR-224 [15]	Low	Tumor suppressor	Rac1

- N/D, not determined; PTEN, phosphatase and tensin homolog; HUWE1; PPARγ, peroxisome proliferator-activated receptor gamma; HES1, hes family bHLH transcription factor 1; MYC, v-myc avian myelocytomatosis viral oncogene homolog ; Bcl-xL, BCL2-like 1 isoform 1; Mcl-1, BCL2 family apoptosis regulator; EZH2, enhancer of zeste 2 polycomb repressive complex 2 subunit; FAS, tumor necrosis factor receptor superfamily member 6; MMP-13, matrix metallopeptidase 13; Bcl-2, B-cell lymphoma 2; ROCK, Rho-associated protein kinase; HMGB1, high mobility group box 1; CCND1, Cyclin D1; MTOR, mechanistic target of rapamycin; Rac1, Ras-related C3 botulinum toxin substrate 1

This is the first meta-analysis that evaluate the prognostic value of miRNAs in osteosarcoma. We demonstrated the association of the outcomes (OS, EFS) of osteosarcoma and level of miRNA expression. In addition, we measured HRs as the effectiveness endpoint in the current study. We did not measured the odds ratios or risk ratios whether these values represent only cross-sectional cumulative estimate and do not including the time factor [35]. Hence, HRs are most appropriate for our report which analyze time-to-event outcomes. The type of samples was homogenous. All of the samples which were experimented in included studies were obtained from tumor tissue of osteosarcoma patients. This might be increased reliability and decreased heterogeneity of our study. We excluded studies using serum or plasma because there were a few studies about serum or circulating miRNAs in patients with osteosarcoma [36, 37]. However, there are increasing interest in circulating miRNAs due to their comfortable accessibility by drawing patient's blood compared to miRNAs of tissue from operation and they could be easily repeated and monitored through.

From the pathway analysis, we found that several common targets and pathways of miRNA199b-5p, 449a and 542-5p, for example, fatty acid biosynthesis (target gene: Fatty Acid Synthase (FASN)) (Table 3). FASN is known as a therapeutic target in the treatment of osteosarcoma metastasis [38]. Pathways related to other malignancies were found in this analysis, such as breast cancer, and prostate cancer [38–41]. The target genes of miRNAs in various cancers may overlap, which suggest that miRNAs could be promising therapeutic targets in cancer treatment.

In this study, we investigated the prognostic value of miRNA in patients with osteosarcoma using a meta-analysis approach. Finally, we concluded that decreased miRNA expression in tumor tissue is associated with worse outcome of patients with osteosarcoma. However, we should also pay attention to increased expression of several miRNAs in tumor tissue of osteosarcoma. Furthermore, prospective studies with a large sample size are warranted to clarify the prognostic role of miRNAs in osteosarcoma.

## MATERIALS AND METHODS

### Data search and study election

We performed a systematic search if MEDLINE (from inception to August 2016) and EMBASE (from inception to August 2016) for English-language publications using the keywords “osteosarcoma”, “miRNA”, “prognosis”. All searches were limited to human studies. The inclusion criteria were studies of osteosarcoma that reported the results of miRNA expressions and survival data. Reviews, abstracts, and editorial materials were excluded. Two authors performed the searches and screening independently, and discrepancies were resolved by consensus.

### Data extraction and statistical analysis

Data were extracted from the publications independently by two reviewers, and the following information was recorded: first author, year of publication, country, miRNA expression analyzed, number of patients, staging, and end points. The primary outcome was OS, which was defined as the time from the initiation of therapy until death from any cause. The secondary end point was EFS. Data regarding disease-free survival (DFS), progression-free survival (PFS), recurrence-free survival (RFS) were obtained from the included studies, and were redefined as EFS, which was measured from the date of initiation of therapy to the date of recurrence or metastasis [42, 43].

The effects of miRNA expressions on survival were assessed using hazard ratios. Survival data were extracted following a methodology suggested previously [44]. A univariate HR estimate and 95% CIs were extracted directly from each study, if provided by the authors. Otherwise, *p* values of the log-rank tests, 95% confidence intervals, number of events, and numbers of patients at risk were extracted to estimate the HR indirectly. Survival rates calculated from Kaplan-Meier curves were read using Engauge Digitizer version 3.0 (http://digitizer.sourceforge.net) to reconstruct the HR estimate and its variance, assuming that patients were censored at a constant rate during follow-up. The HRs were calculated on the basis of low expression of miRNA, which means HR > 1 and < 1 implied poor and good prognosis for patients with low miRNA expression. Heterogeneity among studies was assessed using χ^2^ tests and I^2^ statistics, as described previously [45]. Funnel plots were used to assess publication bias [46]. *p* values < 0.05 were considered to be statistically significant. The data from each study were analyzed using Review Manager (RevMan, Version 5.3 Copenhagen: The Nordic Cochrane Centre, The Cochrane Collaboration, 2014). miRNA target genes and related pathways were predicted by DIANA-mirPath v3.0 (http://www.microrna.gr/miRPathv3) based on Tarbase, KEGG and GO [34] to interpret functional value of miRNAs.
